# A Simpler Draw: Rethinking Colorectal Cancer Screening for Adults With Intellectual and Developmental Disabilities

**DOI:** 10.7759/cureus.115814

**Published:** 2026-09-05

**Authors:** Alison Todd, Raina Modi, Rafik Jacob

**Affiliations:** 1 Department of Medicine, University of Florida, Jacksonville, USA

**Keywords:** blood-based screening, cancer health disparities, colorectal cancer screening, intellectual disability, medicaid coverage

## Abstract

Adults with intellectual and developmental disabilities (IDD) face substantial barriers to colorectal cancer screening, including communication difficulties, caregiver burden, and difficulty completing stool collection or bowel preparation. These barriers likely contribute to lower screening completion and higher cancer-related mortality in this population. Blood-based colorectal cancer screening, which can be completed during a routine blood draw without stool collection or bowel preparation, may reduce several of these logistical barriers. However, most adults with IDD are insured through Medicaid, and current coverage for the leading blood-based test excludes Medicaid beneficiaries in nearly all states, undermining the accessibility argument for this population specifically. We argue that blood-based screening deserves consideration as part of an individualized, equity-focused approach to colorectal cancer prevention in adults with IDD, provided it is offered alongside, not instead of, higher-sensitivity options, and we call for expanded Medicaid coverage and continued improvement in test performance to close the gap between accessibility and effectiveness.

## Editorial

A population left behind

Adults with intellectual and developmental disabilities (IDD) face well-documented disparities in cancer-related outcomes. A Dutch nationwide cohort study found that cancer was reported as the cause of death approximately 1.5 times more often among people with intellectual disability than in the general population, with colorectal cancer accounting for the greatest number of cancer deaths among the malignancies included in the national screening program [1]. These disparities persist despite decades of progress in colorectal cancer prevention. A recent nationwide cohort study found that only 30.2% of people with intellectual disability returned a stool-based screening test, compared with 56.1% of people without intellectual disability [2]. Screening strategies built around independent task completion were never designed with the cognitive, functional, and caregiving realities of this population in mind, and the parallel disparities in screening completion and cancer-related mortality point to the same underlying gap in care design.

Where standard screening falls short

Stool-based testing requires proper collection, handling, and return of an adequate sample, a multistep process complicated by cognitive and functional limitations, communication barriers, sensory sensitivities, and comorbid gastrointestinal motility disorders common in adults with IDD [2]. Even with caregiver support, the completion gap between adults with and without intellectual disability suggests collection is not always completed as intended. Moving collection into a supervised clinic setting does not fully solve the problem, since it still depends on the patient having a bowel movement during the visit and on adequate staffing and time. Colonoscopy introduces a different barrier: bowel preparation, which many adults with IDD cannot understand, tolerate, or complete because of behavioral challenges, inability to consume the prep, or dependence on others for assistance. Inadequate preparation leads to incomplete examinations, reduced diagnostic accuracy, or repeat procedures, and people with intellectual disability have measurably higher rates of nonanalyzable stool samples and incomplete colonoscopy [2]. The barriers compound at nearly every stage of the screening pathway.

What blood-based screening offers

Blood-based colorectal cancer screening has emerged as an additional option for average-risk adults who are unable or unwilling to complete stool-based testing or colonoscopy [3]. This discussion is scoped to adults with IDD who are otherwise appropriate candidates for average-risk screening, since this population is heterogeneous with respect to age, comorbidity, life expectancy, and prior colorectal disease, and the accessibility advantage described here applies within that same average-risk framework rather than to adults with IDD as a single undifferentiated group. Within that group, its appeal is structural rather than diagnostic: it can be incorporated into a routine blood draw already occurring during a clinic visit, without stool collection or bowel preparation. That shifts the burden of completing screening away from the patient and caregiver and into the healthcare setting itself, where it is far more likely to be reliably executed. For patients who face cognitive, functional, communication, or sensory barriers, removing even one multistep task from the process may be the difference between a completed and an abandoned screening.

This advantage comes with a real tradeoff. In the clinical validation study underlying current guideline inclusion, the leading blood-based test demonstrated 83% sensitivity for colorectal cancer, 90% specificity for advanced neoplasia, and only 13% sensitivity for advanced precancerous lesions [3], figures that fall short of stool-based and endoscopic alternatives. A positive result still requires diagnostic colonoscopy [3]. Blood-based screening is not a substitute for preferred methods when those methods are feasible and acceptable to the patient. Its value for this population lies specifically in reaching patients who would otherwise complete no screening at all.

This limitation carries particular weight for adults with IDD, since the same barriers that make bowel preparation and colonoscopy difficult in the first place, discussed above, do not disappear at the diagnostic step. Improving initial screening uptake through a blood draw could simply relocate the point of failure rather than resolve it: a patient who completes blood-based screening but cannot complete the subsequent colonoscopy remains without a diagnosis, and without the benefit screening is meant to provide. The accessibility advantage of blood-based screening is therefore contingent on parallel investment in supporting colonoscopy completion after a positive result, including advance planning for anesthesia, adapted bowel-prep protocols, and caregiver coordination specific to this population. Absent that investment, blood-based screening risks shifting the diagnostic-completion gap downstream rather than closing it.

A coverage gap that undercuts the case

The population most likely to benefit from a lower-burden screening option is also the population least likely to have it covered. Adults with IDD rely disproportionately on Medicaid for health coverage: in a national analysis of adults with IDD enrolled in Medicare, three-quarters were also dually enrolled in Medicaid [4], and Medicaid-only enrollment accounts for a substantial share of adult IDD coverage beyond that. Yet the test manufacturer's own billing data indicate that, as of March 2026, the leading blood-based colorectal cancer screening test was covered under Medicaid in only two states (Florida and Kentucky) and not covered in the remaining 48 [5]. No independent state-level Medicaid tracking currently exists for this specific test to confirm the picture beyond that source. An option promoted for removing logistical barriers is, for most of the population this argument is built around, not actually reachable because of cost. Closing this gap is a policy and advocacy problem as much as a clinical one. Clinicians caring for adults with IDD have a role in pressing state Medicaid programs and payers to extend coverage to match what Medicare already provides, so that accessibility on paper becomes accessibility in practice.

Guardrails: accessibility is not a lower standard

Greater accessibility must not become a rationale for offering a lesser standard of care. A diagnosis of IDD should not, by itself, determine which screening test a patient is offered. Current American Cancer Society guidance lists blood-based testing as an acceptable screening option but recommends it only for individuals who decline or do not complete preferred stool-based or endoscopic methods [3]. Adults with IDD should still be offered the full range of appropriate screening options, with caregivers and guardians supporting decision-making where appropriate, so that any choice of blood-based testing is an informed one rather than a default made for convenience. That conversation should include the test's lower sensitivity and the near-certain need for a follow-up colonoscopy if the result is positive. Medicare currently covers qualifying blood-based colorectal cancer screening every three years for average-risk, asymptomatic adults [3]; extending comparable coverage through Medicaid would remove one of the largest practical barriers to offering this option equitably.

The path forward

The open question is not whether blood-based screening performs as well as colonoscopy; it does not. The open question is whether it increases the number of adults with IDD who complete any colorectal cancer screening at all, and whether that increase translates into timely diagnostic follow-up after a positive result. Future work should track not just screening completion but colonoscopy completion, time to follow-up, cancer detection, and downstream outcomes in this population, along with the role of caregiver and provider education and electronic health record reminders in supporting the pathway once a patient screens positive. We are hopeful that ongoing advances in blood-based screening technology, including improvements in assay sensitivity for early-stage cancer and advanced precancerous lesions, will narrow the performance gap with colonoscopy over time. A test that is both accessible and highly sensitive would resolve the tradeoff this editorial has had to work around, and continued investment in that direction deserves support alongside advocacy for broader Medicaid coverage. Colorectal cancer prevention has not yet caught up to the needs of adults with IDD, on either front.
